# Epoxygenase inactivation exacerbates diet and aging-associated metabolic dysfunction resulting from impaired adipogenesis

**DOI:** 10.1016/j.molmet.2018.03.003

**Published:** 2018-03-09

**Authors:** Antoni Olona, Ximena Terra, Jeong-Hun Ko, Carme Grau-Bové, Montserrat Pinent, Anna Ardevol, Ana Garcia Diaz, Aida Moreno-Moral, Matthew Edin, David Bishop-Bailey, Darryl C. Zeldin, Timothy J. Aitman, Enrico Petretto, Mayte Blay, Jacques Behmoaras

**Affiliations:** 1 Centre for Complement and Inflammation Research, Imperial College London, London, W12 0NN, UK; 2 Mobiofood Research Group, Department of Biochemistry and Biotechnology, Universitat Rovira i Virgili, 43007, Tarragona, Spain; 3 Renal and Vascular Inflammation Section, Department of Medicine, Imperial College London, London, W12 0NN, UK; 4 Duke-NUS Medical School, National University of Singapore, 169857, Singapore; 5 Division of Intramural Research, National Institute of Environmental Health Sciences, National Institutes of Health, Research Triangle Park, NC, USA; 6 Comparative Biomedical Sciences, Royal Veterinary College, London, NW1 0TU, UK; 7 Centre for Genomic and Experimental Medicine, Institute of Genetics and Molecular Medicine, University of Edinburgh, Edinburgh, UK

**Keywords:** Adipogenesis, Cytochrome P450 2j4, Cafeteria diet, Aging, Steatosis, Arachidonic acid, WAT, white adipose tissue, PPAR-γ, peroxisome proliferator-activated receptor-γ, C/EBPα, CCAAT/enhancer binding protein-α, ECM, Extracellular Matrix, NAFLD, Non-alcoholic fatty liver diseases, Cyp, cytochrome P450, sEH, epoxide hydrolase, CAF, Cafeteria diet, EET, Epoxyeicosatrienoic acid, COX, Cyclooxygenase, LOX, Lipoxygenase, MSC, Mesenchymal Stromal Cells, SVF, Stromal Vascular Fraction, *FASN*, Fatty acid synthase gene

## Abstract

**Objective:**

When molecular drivers of healthy adipogenesis are perturbed, this can cause hepatic steatosis. The role of arachidonic acid (AA) and its downstream enzymatic cascades, such as cyclooxygenase, in adipogenesis is well established. The exact contribution of the P450 epoxygenase pathway, however, remains to be established. Enzymes belonging to this pathway are mainly encoded by the *CYP2J* locus which shows extensive allelic expansion in mice. Here we aimed to establish the role of endogenous epoxygenase during adipogenesis under homeostatic and metabolic stress conditions.

**Methods:**

We took advantage of the simpler genetic architecture of the *Cyp2j* locus in the rat and used a *Cyp2j4* (orthologue of human *CYP2J2*) knockout rat in two models of metabolic dysfunction: physiological aging and cafeteria diet (CAF). The phenotyping of *Cyp2j4*^*−/−*^ rats under CAF was integrated with proteomics (LC-MS/MS) and lipidomics (LC-MS) analyses in the liver and the adipose tissue.

**Results:**

We report that *Cyp2j4* deletion causes adipocyte dysfunction under metabolic challenges. This is characterized by (i) down-regulation of white adipose tissue (WAT) PPARγ and C/EBPα, (ii) adipocyte hypertrophy, (iii) extracellular matrix remodeling, and (iv) alternative usage of AA pathway. Specifically, in *Cyp2j4*^−/−^ rats treated with a cafeteria diet, the dysfunctional adipogenesis is accompanied by exacerbated weight gain, hepatic lipid accumulation, and dysregulated gluconeogenesis.

**Conclusion:**

These results suggest that AA epoxygenases are essential regulators of healthy adipogenesis. Our results uncover their synergistic role in fine-tuning AA pathway in obesity-mediated hepatic steatosis.

## Highlights

• *Cyp2j4* deletion in rats offers a simplified genetic landscape as opposed to the mice locus, which is under significant allelic expansion.
• Under different metabolic stresses (aging, cafeteria diet), *Cyp2j4*^−/−^ rats show an accelerated adipogenesis, which progress towards adipocyte dysfunction.
• Adipocyte dysfunction in *Cyp2j4*^*−/−*^ rats under cafeteria diet (CAF) is characterised by down-regulation of white adipose tissue (WAT) PPARγ and C/EBPα, adipocyte hypertrophy and extracellular matrix remodeling.
• *Cyp2j4*^−/−^ rats treated with CAF display exacerbated weight gain, insulin resistance, hepatic lipid accumulation and dysregulated gluconeogenesis.
• *Cyp2j4*^*−/−*^ rats display alternative arachidonic acid pathway usage in their adipose tissue upon CAF and aging.

## 1 Introduction

Obesity is a complex metabolic disorder with complications such as insulin resistance, chronic inflammation, and hepatic steatosis, all of which under the influence of white adipose tissue (WAT), a highly dynamic, master-regulatory endocrine organ crucial for metabolic homeostasis [1], [2]. It is argued that the key mediators of obesity-mediated metabolic disease (i.e. insulin resistance and inflammation) are evolutionarily conserved but could display pathological properties under modern obesogenic environment, which is characterized by excess nutrient consumption [3].

At the heart of WAT homeostasis, adipogenesis is the process of differentiation of pre-adipocytes to become mature under a core transcriptional program driven by nuclear hormone receptor peroxisome proliferator-activated receptor-γ (PPAR-γ) and CCAAT/enhancer binding protein-α (C/EBPα) [4], [5]. In addition to the role of PPAR-γ in embryonic adipogenesis, C/EBPα and PPAR-γ are actively involved in adult WAT expansion following high dietary fat exposure [6]. During healthy WAT expansion, hypoxia and inflammation caused by activated macrophages lead to extracellular matrix (ECM) remodeling, which enables adipocyte hypertrophy [7]. However, in the case of chronic over-nutrition, this state of homeostasis is perturbed and causes unresolved, low grade WAT inflammation and fibrosis. The fibrotic and unrestrained WAT expansion, often promoted by pro-inflammatory macrophage activity, can eventually progress into adipose tissue dysfunction and ectopic lipid accumulation, in particular in the liver, one of the major contributors of obesity-mediated type 2 diabetes [1]. Non-alcoholic fatty liver disease (NAFLD) is characterized by hepatic lipid accumulation, which could lead to inflammation and fibrosis in the liver. The central role of adipose tissue in the development of NAFLD was established in humans [8], [9] and animal models of diet-induced obesity [10]. Aging is also considered as a risk factor for insulin resistance and adipose tissue plays a central role in longevity. Aging and diet-induced obesity share pathways including WAT-mediated lipotoxicity [11], which suggests that common genes orchestrate WAT homeostasis and its regulatory role on NAFLD.

Oxylipins are endogenous, bioactive lipid mediators derived from arachidonic acid (AA) and related polyunsaturated fatty acids. Prostaglandins and leukotrienes are eicosanoids generated by well-defined enzymatic cascades initiated by cyclooxygenase and lipoxygenase [12]. A third pathway involves cytochromes P450 (CYPs). In humans, cytochrome P450 2J2 (CYP2J2), CYP2C8, and CYP2C9 are considered to be largely responsible for metabolizing AA into four regioisomeric epoxyeicosatrienoic acids (5,6-, 8,9-, 11,12-, and 14,15-EET) [13]. EETs are metabolized by soluble epoxide hydrolase (sEH) to the corresponding dihydroxyeicosatrienoic acids and sEH inhibition is a commonly used pharmacological approach aimed to increase intracellular EET pools. The previously reported biological effects of EETs are remarkably pleiotropic, ranging from anti-inflammatory and cardioprotective actions [14], [15], [16] to a regulatory role in cancer [17], organ/tissue regeneration [18], and embryonic haematopoiesis [19].

EETs are PPARγ ligands [20] and activators of PPARα [21]. Given the central role exerted by PPARγ in regulating adipogenesis, the link between epoxygenase-mediated EET production and obesity-associated syndromes was explored in transgenic animal models over-expressing human endothelial CYP2J2 or by inactivation of sEH (either by pharmacological inhibition or its targeted gene deletion [22], [23], [24], [25]). These studies, which aimed to increase endogenous EET levels, achieved amelioration of obesity-associated metabolic dysfunction (i.e. dyslipidemia, prevention of hyperglycemia, improved insulin signaling and sensitivity, reduced AT inflammation). However the exact mechanisms through which the main endogenous epoxygenase regulate metabolic dysfunction remain poorly understood, mainly because of the technical obstacle encountered in gene targeting approaches in mice. The *Cyp2j* locus in mice contains eight potentially functional genes as it underwent allelic expansion [26]. The synthenic rat *Cyp2j* locus contains three genes and offers a relatively simplified genetic architecture for studying epoxygenase-related mechanisms. Thus, we have generated a rat deficient in *Cyp2j4*, the orthologue of human *CYP2J2* [27]. *Cyp2j4* is the main rat macrophage epoxygenase, which also shows wide-tissue expression including brain, left ventricle, kidney, lung, and spleen [27]. Although *Cyp2j3* (LOC100912642, cytochrome P450 2J3-like) maps to rat chromosome 5 and was initially reported as the rat orthologue of human *CYP2J2* [28], both genes were found to be expressed in major rat organs and share 79% homology.

Here we took advantage of the reduced allelic expansion in the rat *Cyp2j* locus and used two distinct models of metabolic dysfunction to study epoxygenase-mediated adipogenesis in the wider context of obesity and NAFLD. In addition to physiological aging, we used a Western diet-induced obesity model, previously described as cafeteria diet (CAF), which takes into account hedonic feeding (or voluntary hyperphagia) [29]. We have previously shown strain-specific differences in CAF-induced metabolic dysfunction in the rat [30], [31]. We report that *Cyp2j4* is essential for maintaining a healthy adipogenesis status, which, under metabolic challenges (e.g. CAF, aging), causes adipocyte dysfunction characterized by down-regulation of WAT PPARγ and C/EBPα and AA pathway shunt towards COX and LOX-derived eicosanoids. This dysfunctional adipogenesis causes hepatic lipid accumulation and *Cyp2j4*^−/−^ treated with CAF show increased *de novo* lipogenesis in the liver, dysregulated gluconeogenesis, and increased hepatic and systemic triglyceride levels. These results determine the role of *Cyp2j4* in physiological (healthy) adipogenesis and show how this ‘controlled’ phenomenon progresses into adipocyte dysfunction and NAFLD under metabolic stresses such as CAF and aging.

## 2 Materials and methods

### 2.1 Animals

Male wild type Wistar Kyoto (WKY) rats (Charles River, UK) and *Cyp2j4*^−/−^ rats, previously generated on a WKY genetic background [27] were housed individually at 22 °C with a 12 h light/dark cycle with access to water and a standard diet *ad libitum*. The animals were maintained according to the ethical guidelines of Universitat Rovira i Virgili (URV, Committee on Animal Investigations) or the UK Home Office (United Kingdom Animals Scientific Procedures Act, 1986).

### 2.2 Cells and reagents

Mesenchymal stromal cells (MSCs) from 12-week old WT and *Cyp2j4*^−/−^ rats were obtained as previously described [32]. MSCs cells were allowed to grow in Supplemented MesenCult™ MSC Medium (STEMCELL Technologies, UK) for 5 days on Petri dishes (Nunc, ThermoFisher Scientific, UK). MSCs from WT and *Cyp2j4*^−/−^ rats were differentiated into mature adipocytes by incubation with an adipogenic induction medium (StemPro®,Gibco, UK) for 14 days.

Antibodies used in western blot were: anti-PPAR*-γ* (C26 H12, Cell Signaling #2435, 1:1000), anti CEBP*-α* (Cell Signaling #2295, 1:1000), anti-Phospho-Akt-Ser473 (D9E, Cell Signaling #4060, 1:2000), anti-Phospho-Akt-Thr308 (244F9, Cell Signaling #4056, 1:1000), anti-panAkt (C67E7, Cell Signaling #4691, 1:1000) and anti-β-Actin Antibody (C4, sc-47778, 1:10,000), anti-PPARα (H2, SC-398,394, 1:1000), anti-PPARβ/δ (F-10, SC-74517, 1:1000), anti-FXR (D-3, SC-25309, 1:1000), anti-LXRα (ab2585, 1:1000), and anti-β-Actin Antibody (C4, sc-47778, 1:10,000).

### 2.3 Cafeteria diet and aging

Eight-week-old WT and *Cyp2j4*^−/−^ rats were randomly distributed into the four different experimental groups to receive either a standard laboratory chow (STD, A-04; Panlab) or a standard laboratory chow together with cafeteria diet (CAF) consisting of 300 ml of sugary milk (220 g/L), 25 g of bacon, 1 sausage, ¼ carrot and 2 biscuits smeared with paté. Both WT and *Cyp2j4*^−/−^ rats were fed either with standard diet (WT STD, *Cyp2j4*^−/−^ STD) or CAF (WT CAF, *Cyp2j4*^−/−^ CAF). Animals were fed *ad libitum* with fresh food daily for 12 weeks. For the aging protocol, WT and *Cyp2j4*^−/−^ rats received a standard laboratory chow during 15 months. At the end of both CAF and aging protocols, plasma was collected from all animals and kept for biochemistry analysis. A section of the liver and skin were kept in formalin for further immunohistochemical studies. Retroperitoneal, mesenteric, epididymal, and subcutaneous white adipose tissues, were isolated, weighed, and kept in formalin for immunohistochemistry. Sections from the liver and adipose tissue were frozen in optical coherence tomography (OCT) solution at −80 °C for Oil-Red-O staining and immunofluorescence. To isolate the stromal vascular fraction (SVF) from the adipose tissue following CAF, the tissue was first washed to remove red blood cells, cut in small pieces and incubated for 45 min with collagenase P (Worthington-Biochem, USA) in HBSS at 37 °C. The digested adipose tissue was then passed through a sterile strainer (70 μm porus diameter), washed three times in PBS, incubated with red blood cell lysis solution. Following CAF, frozen liver and SVF fractions were kept for quantitative proteomics by LC-MS/MS. The morphometric and biochemical measurements, food intake, as well as adipocyte size and volume quantification are detailed in Supplementary methods A.

### 2.4 Western blotting

Protein lysates from liver and epididymal white adipose tissue (WAT) were homogenized in RIPA buffer (Sigma–Aldrich) supplemented with 1% protease inhibitor Cocktail (Thermo Fisher Scientific). Lysates were centrifuged for 10 min, 10,000 g, at 4 °C and supernatants were used for Western blot analysis. Total protein concentration was determined by Bicinchoninic Acid Kit for Protein Determination (Thermo Fisher Scientific). 20 μg of total cellular protein was diluted 1:1 with 2x Laemmli buffer (Bio-Rad) and denatured at 95 °C for 5 min. MSC protein lysates were directly homogenised in 2x Laemmli buffer (Bio-Rad). Final protein lysates were resolved by SDS-PAGE 7% and transferred to PVDF membranes in 20% methanol, 200 mM Gly, 25 mM Tris, pH 8.3. The membrane was blocked for 1 h at room temperature and then incubated overnight at 4 °C with primary antibodies. The blots were washed and exposed to horseradish peroxidase-labeled secondary antibody (1:10,000) for 1 h at room temperature. The blots were then washed and the immunocomplexes visualized by the chemiluminescence detection system SuperSignal West Pico PLUS Substrate (Thermo Fisher Scientific).

### 2.5 Quantitative Reverse Transcription PCR

Total RNA from liver, adipose tissue, and MSCs was extracted using Trizol (Ambion) according to the manufacturer's instructions. Complementary DNA (cDNA) was obtained from 1 μg of mRNA using the Bio-Rad iScript kit (Bio-Rad, UK) according to the manufacturer's instructions. Quantitative Reverse Transcription PCR *(*qRT-PCR) reactions were performed using the Viaa 7 Real-Time PCR system (Life technologies). A total of 10 ng of cDNA per sample was used for PCR using Brilliant II SYBR Green QPCR Master Mix (Agilent). Viia 7 RUO Software was used for the determination of Ct values. Results were analyzed using the comparative Ct method and each sample was normalized to the reference gene (Hprt or Ppia) to account for any cDNA loading differences. The forward and reverse primer sequences used are provided in Supplementary methods A.

### 2.6 Immunohistochemistry and immunofluorescence

For rat ED-1 (CD68) immunostaining, 3 μm thick paraffin adipose tissue sections were prepared using a Microm HM 440E (Thermo Fisher Scientific, Waltham, MA, USA) and blocked with 3% bovine serum albumin (BSA; Sigma–Aldrich) for 1 h at room temperature. The sections were then incubated with primary antibodies against ED-1 (Bio-Rad AbD Serotec, UK) in phosphate-buffered saline (PBS) and supplemented with 3% BSA overnight at 4 °C. The sections were washed in PBS, followed by the incubation with horseradish peroxidase-labeled secondary antibody (1:10,000) for 1 h at room temperature (DAKO EnVision™+ System; Agilent Technologies (UK)). Diaminobenzidine (DAB) chromogen was then added, and the slides were visualized using an Olympus BX40 microscope (Olympus, UK) equipped with a digital camera Retiga 2000R CCD (QImaging, Canada). Pictures were further analyzed using ImageJ software. For immunofluorescence, adipose tissue and liver samples were fixed in 10% formalin overnight, processed and embedded in paraffin blocks. 5 μm thick sections were placed onto microscope slides, dewaxed and rehydrated. Antigen retrieval with sodium citrate buffer (pH 6) was carried out prior to blocking. Slides were then incubated overnight with Goat Anti-Type I Collagen (1310-01) or Goat Anti-Type VI Collagen (1360-01) from Southern Biotech (Birmingham, USA). After washing, slides were incubated 1 h with Donkey Anti-Goat Alexa Fluor 488 (ab150129, Abcam, Cambridge, UK). Slides were mounted using VECTASHIELD medium. Images were taken using epi-fluorescent Leica DM4B microscope and the raw fluorescence intensity was acquired using the ImageJ software.

### 2.7 Lipid oil-red-O staining and TAG quantification

Frozen rat liver tissues and formalin (10%) fixed MSCs were used for Oil Red O staining (0.5% Oil Red O dye in isopropanol; Sigma) and H&E and photographed by light microscopy (Olympus BX40 microscope). For the MSCs, the ratio of oil red o + red area to total area in each microscopic field was calculated by the ImageJ software.

For TAG quantification, liver tissue samples were homogenized in 5% NP-40 in ddH_2_O. Samples were progressively heated to reach 100 °C and left to cool down. After repeating this process, samples were centrifuged to remove insoluble material. Total TAG content was quantified using Infinity Triglycerides Liquid Stable Reagent (Sigma) following the manufacturer's instructions.

### 2.8 Quantitative proteomics by LC-MS/MS

Liver, AT stromal vascular fraction (SVF), and BMDM lysates containing a total protein amount of 200 μg in 8 M urea and 20 mM HEPES buffer (pH 8.0) were reduced and alkylated sequentially with 10 mM Dithiothreitol and 50 mM Iodoacetamide, respectively. Trypsin Gold (Promega, V5280) was added into the diluted samples (2 M Urea) to reach a final protease to protein ratio of 1:50. Samples were incubated overnight at 37 °C, acidified with trifluoroacetic acid (TFA) and de-salted using solid phase extraction (Waters OASIS HLB 10 mg cartridges) according to the manufacturer's instructions. Eluents were vacuum centrifuged to dryness.

Samples were re-dissolved in 0.1% TFA (200 μl/sample) by shaking (1200 rpm) for 30 min and sonicated in an ultrasonic water bath for 10 min, followed by centrifugation (14,000 rpm, 4 °C) for 10 min. LC-MS/MS analysis was carried out in technical duplicates. Peptides were first separated using an Ultimate 3000 RSLC nano liquid chromatography system (Thermo Scientific) coupled to a Q-Exactive mass spectrometer (Thermo Scientific) via an EASY-Spray source. For LC-MS/MS analysis, sample volumes containing 1.0 μg of total tryptic digest were injected and loaded onto a trap column (Acclaim PepMap 100 C18, 100  μm × 2 cm) for desalting and concentration at 8 μL/min in 2% acetonitrile, 0.1% TFA. Peptides were then eluted on-line to an analytical column (Acclaim Pepmap RSLC C18, 75  μm × 25 cm). Peptides were separated using a linear 120 min gradient, 4–45% of buffer B (composition of buffer B – 80% acetonitrile, 0.1% formic acid), and eluted peptides were analyzed by the Q-Exactive operating in positive polarity using a data-dependent acquisition mode. Ions for fragmentation were determined from an initial MS1 survey scan at 70,000 resolution (at *m*/*z* 200), followed by higher-energy collisional dissociation of the top 12 most abundant ions at a resolution of 17,500. MS1 and MS2 scan AGC targets were set to 3e6 and 5e4 for a maximum injection times of 50 ms and 100 ms respectively. A survey scan *m*/*z* range of 400–1600 *m*/*z* was used, with a normalised collision energy set to 28%, underfill ratio – 2%, charge state exclusion enabled for unassigned, +1, +6–8 and >+8 ions.

Data were processed using the MaxQuant software platform (v1.5.6.0), with database searches carried out by the in-built Andromeda search engine against the Uniprot rattus norvegicus _20,170,214 database (Downloaded – 2nd February 2017version 20,170,214, number of entries: 35,839). A reverse decoy database approach was used at a 1 & 5% false discovery rate (FDR) for peptide spectrum matches and protein identification. Search parameters included: maximum missed cleavages set to 2, fixed modification of cysteine carbamidomethylation and variable modifications of methionine oxidation, protein N-terminal acetylation, Asparagine deamidation, and cyclization of N-terminal glutamine to pyroglutamate. Label-free quantification was enabled with an LFQ minimum ratio count of 2. ‘Match between runs’ function was used with match and alignment time limits of 2 and 20 min respectively. The LC-MS/MS differential protein analysis is in Supplementary methods A.

### 2.9 Lipidomics by LC-MS

Frozen WAT (50 mg) was homogenized in 400 μl ice-cold methanol with 0.1% acetic acid and internal standard for 10 min, centrifuged at 10,000 rpm, 10 min at 4 °C, and the pellets were re-extracted with 100 μl of ice-cold methanol containing 0.1% of acetic acid. The supernatants were spiked with internal standard [3 ng PGE_2_-d4, 11,12-EET-d11 and 11,12-DHET-d11, (Cayman Chemical, Detroit, MI), combined with 2 ml of water and shaken. Following serial passage through HyperSep Retain SPE columns (Thermo Scientific, Bellefonte, PA), the columns were washed and then eluted with 0.5 ml of methanol and 1 ml of ethyl acetate into glass tubes containing 10 μl of glycerol (30%) in methanol. The eluates were dried under vacuum centrifugation and reconstituted in 50 μl of ethanol (30%).

Eicosanoid extraction was performed as previously described [33]. Briefly, online LC of extracted samples was performed with an Agilent 1200 series capillary HPLC (Agilent Technologies, Santa Clara, CA). Separations were achieved using a Halo C18 column (2.7 um, 100 × 2.1 mm; MAC-MOD Analytical, Chadds Ford, PA). Electrospray ionization MS/MS was performed on an MDS Sciex API 3000 equipped with a TurboIonSpray source (Applied Biosystems, Foster City, CA). The relative response ratios was calculated based on a curve of known standards (Cayman Chemical) with correction for recovery of internal standards using Analyst 1.5.1 software (Applied Biosystems). Eicosanoid concentrations were normalized to tissue weight. Adipose tissue eicosanoid levels in WAT were represented in a heatmap (see Supplementary Figure A. 5B), where rows (z-scores) were clustered by using correlation distance measure from Heatmap function in XLStat 19.5 (Addinsoft) software. Non-specific filtering was used to remove the features with low variability (interquartile range < 0.25) prior to analyses.

### 2.10 Statistical analysis

Results are expressed as the mean ± SEM and were analyzed using GraphPad Prism 6.0 software (GraphPad, USA). All statistical analyses were performed with Student's t-test or ANOVA. Statistical analysis of LC-MS/MS data is detailed in Supplementary methods A.

### 2.11 Data availability

The rat macrophage RNA-seq data is available at the National Center for Biotechnology Information's Gene Expression Omnibus (http://www.ncbi.nlm.nih.gov/geo/) under accession number GSE65715. The LC-MS/MS Maxquant as well as lipidomics (LC-MS) data are available upon request.

## 3 Results

### *3.1* *Cyp2j4* deletion causes enhanced adipogenesis and weight gain during aging

**Figure 1:**
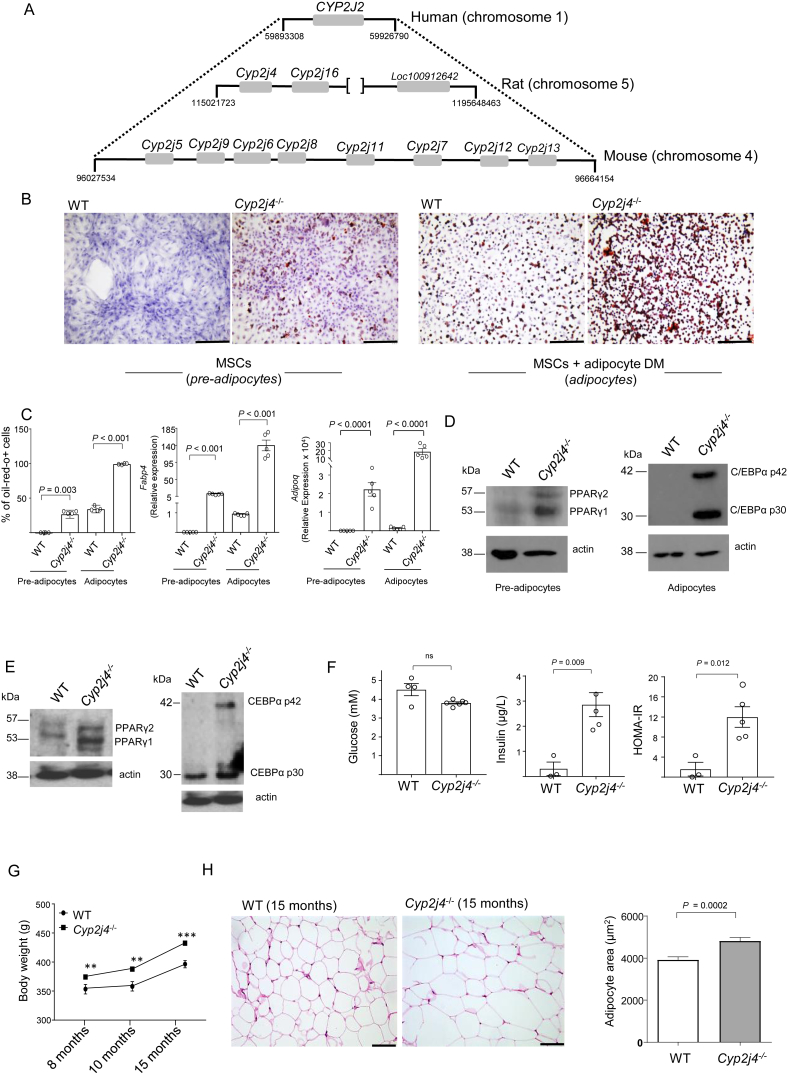
*Cyp2j4*^−/−^ mesenchymal stromal cells (MSCs) undergo spontaneous adipogenesis, and aging *Cyp2j4*^−/−^ rats show increased body weight and larger adipocytes. (A) The genomic synteny between human *CYP2J2* (reference) locus and the corresponding rat and mice loci. [ ] denote the interruption in the genomic distance scale; positions are in bp. (B) Oil-red-o (ORO) staining in MSCs from WT and *Cyp2j4*^−/−^ before (left panel, pre-adipocytes) and after (right panel, adipocytes) adipocyte differentiation with the addition of adipogenic differentiation medium (DM). (C) ORO staining quantification as well as *Fabp4* and *Adipoq* gene expression in MSC-derived pre-adipocytes and adipocytes. (D) Western blot analysis of PPARγ (pre-adipocytes) and C/EBPα (adipocytes) in *Cyp2j4*^−/−^ and WT cells. Blots are representative of 2 independent experiments. (E) Western blot analysis of PPARγ and C/EBPα in the adipose tissue from WT and *Cyp2j4*^−/−^ rats. (F) Glucose, insulin levels, and HOMA-IR index in 15-month old WT (n = 4) and *Cyp2j4*^−/−^ (n = 6) rats' plasma (G) Body weight evolution in aging WT (n = 4) and *Cyp2j4*^−/−^ (n = 6) rats under standard chow diet. (H) Representative Haematoxylin and Eosin (H&E) white adipose tissue (WAT) staining (left panel) and adipocyte area quantification in 15-month old WT and *Cyp2j4*^−/−^ rats. Error bars are s.e.m. Scale bars; 250 μm (B); 100 μm (H).

The CYP2J locus containing *CYP2J2* in humans and its synteny with mice and rats shows a reduced allelic expansion in the rat, suggesting that the rat (Figure 1A) is a more appropriate model to study the role of P450 epoxygenases in adipogenesis and metabolic syndrome. Mesenchymal stromal cells (MSCs) have been largely described for their multipotent capacity to differentiate into osteoblasts, chondrocytes, and adipocytes *in vitro* and *in vivo* [34]. Here we cultured primary bone marrow derived MSCs from WT and *Cyp2j4*^−/−^ rats and observed spontaneous adipogenesis (i.e. without the addition of the adipogenic differentiation media) in *Cyp2j4*^−/−^ MSCs, showing 26.78% oil-red-o positivity and significantly higher *Fabp4* and *Adipoq* expression (Figure 1B–C). The addition of adipocyte differentiation media stimulated adipogenesis on a relatively faster rate in *Cyp2j4*^−/−^ MSCs (Figure 1B–C). In non-differentiated MSCs (designated as pre-adipocytes), the *Cyp2j4* deletion is associated with increased PPARγ levels (Figure 1D). Because C/EBPα is induced at later stages and is active in mature adipocytes [6], [35], we tested whether C/EBPα levels were under the control of *Cyp2j4* and found up-regulation of C/EBPα protein levels in the absence of *Cyp2j4* (Figure 1D). In addition to the modulation of adipogenesis, recent studies identified PPARγ as an important regulator of extracellular matrix homeostasis [36]. In line with the overall reduced extracellular matrix (ECM) remodeling during adipogenesis [37], [38], we found that in pre-adipocytes and differentiated adipocytes, *Cyp2j4* deletion resulted in a significant reduction in type I, III, and VI collagen expression levels, suggesting its regulatory role on collagens involved in WAT expansion (Supplementary Figure A.1). Based on the *in vitro* results showing the regulatory role of *Cyp2j4* in adipogenesis, we hypothesized a wider *in vivo* metabolic effect of this epoxygenase. Accordingly, PPARγ and C/EBPα were found to be up-regulated in *Cyp2j4*^−/−^ WAT (Figure 1E). Furthermore, 15-month-old *Cyp2j4*^*−/−*^ rats showed an altered systemic glycemic profile (Figure 1F) and, when body weight was monitored monthly in aging rats on a standard chow diet, we observed a significant weight gain (Figure 1G) and significantly increased adipocyte hypertrophy in *Cyp2j4*^*−/−*^ rats when compared with the WT controls (Figure 1H).

### 3.2 Epoxygenase-mediated metabolic dysfunction and adipocyte hypertrophy upon CAF

**Figure 2:**
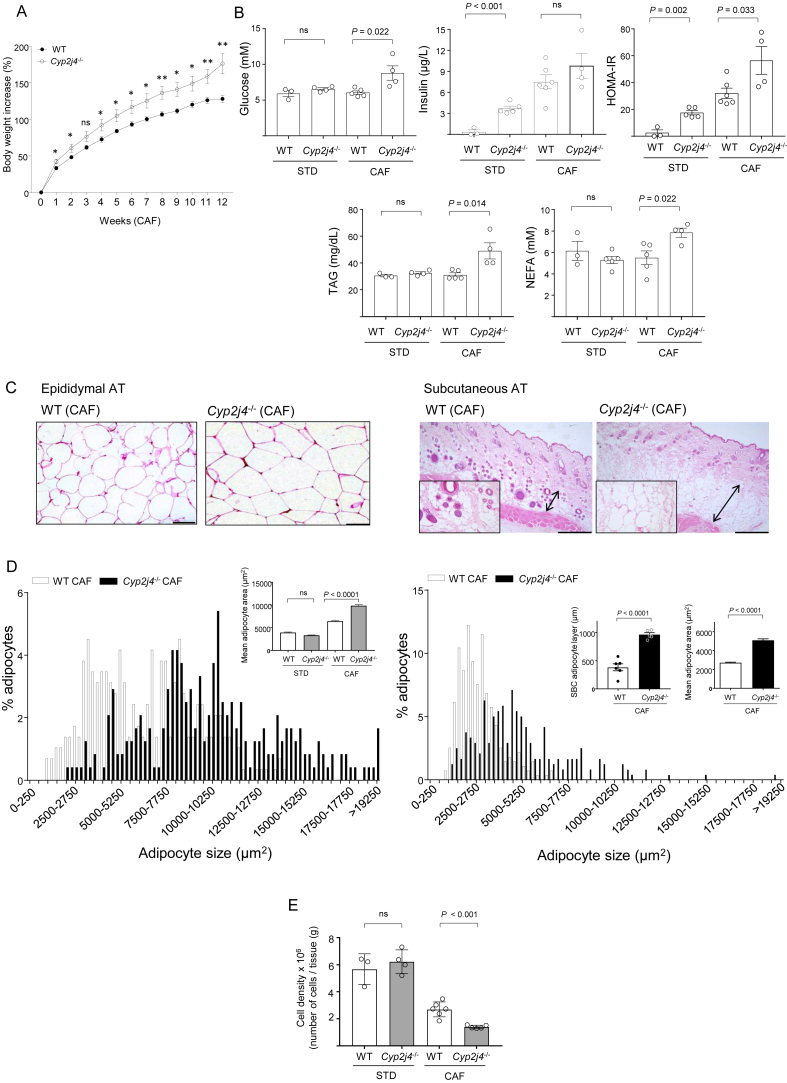
*Cyp2j4*^−/−^ rats show relatively increased metabolic dysfunction under CAF. (A) Percentage of body weight gain in WT (n = 5) *Cyp2j4*^−/−^ (n = 4) rats during 12 weeks of CAF. (B) Glucose, insulin, HOMA-IR, TAG, and NEFA levels measured in WT and *Cyp2j4*^−/−^ rats' plasma upon standard (STD) or cafeteria diet (CAF). At least n = 3 rats were used in each group. (C) Representative H&E staining in epididymal (left panel) and subcutaneous fat (right panel) sections. The arrows indicate the thickness of the subcutaneous adipose tissue layer and its larger magnification (×40) is shown at bottom left. (D) Adipocyte area distributions in WT CAF (open bars) and *Cyp2j4*^−/−^ CAF (black bars) where adipocytes are grouped into ascending sizes of 250 μm^2^ (size range 250 μm^2^) in epididymal (left panel) and subcutaneous fat (right panel). For clarity, 9 group sizes are shown in the x-axis. Mean adipocyte area is shown for all groups (epididymal AT, top left). Subcutaneous (SBC) adipocyte layer and mean adipocyte layer are shown for WT (CAF) and *Cyp2j4*^−/−^ rats (subcutaneous AT, top right). (E) WAT cell density in WT and *Cyp2j4*^−/−^ rats in STD diet and CAF. At least n = 3 rats were used in each group. ns, non-significant. Scale bars, 100 μm (epididymal AT) and 500 μm (subcutaneous AT).

The spontaneous adipogenesis in *Cyp2j4*^−/−^ MSCs led us hypothesize that the deletion of *Cyp2j4* could cause metabolic dysfunction upon treatment with CAF. To test this, WT and *Cyp2j4*^−/−^ rats were subjected to either a standard (STD) or CAF, and body, WAT weights, as well as the percentage of adiposity were measured. The energy intake was not found to be different between the WT and *Cyp2j4*^−/−^ animals under the CAF (Supplementary Figure A.2). As expected, animals that received a CAF had increased body weight, WAT weight, and percentage of adiposity when compared with those treated with a STD diet (Table 1). *Cyp2j4*^−/−^ (CAF) rats showed higher percentage of body weight increase, adiposity, and WAT weight than the WT (CAF) controls (Table 1). Throughout the 12-weeks of CAF treatment, *Cyp2j4*^−/−^ rats showed a significantly higher percentage of weight increase, from the first week onwards, reaching the strongest difference with WT animals at week 12 (Figure 2A).

**Table 1:** Morphometric variables of WT and *Cyp2j4*^−/−^ rats under a standard (STD) or cafeteria (CAF) diet.

*Morphometric variables*	WT (STD)	***Cyp2j4***^−/−^**(STD)**	WT (CAF)	***Cyp2j4***^−/−^**(CAF)**
Body weight (g)	341.3 ± 19.7	326.4 ± 7.1	398.7 ± 3.7*	429.4 ± 16.8*
WAT weight (g)	14.3 ± 0.7	13.9 ± 0.7	25.6 ± 1.7*	37.7 ± 1.86*^,#^
% Adiposity	4.2 ± 0.1	4.3 ± 0.2	6.4 ± 0.4*	8.8 ± 0.2*^,#^
% Body weight gain	111.4 ± 12.2	114.5 ± 8.1	128.1 ± 5.5	176.4 ± 14.2*^,#^

Values are means ± SEM. *, *P* < 0.05, Comparison between diets (CAF vs STD). #, *P* < 0.05, Comparison between strains (*Cyp2j4*^−/−^ vs. WT).

In addition to morphometric variables, we examined parameters related to the metabolic state of the animals after the CAF. For this purpose, the glycemic profile (glucose, insulin, and HOMA-IR index) and lipid levels (triacylglyceride or TAGs; non-esterified fatty acids or NEFAs) were measured in all groups (Figure 2B). *Cyp2j4*^−/−^ and WT rats under a STD diet showed significant differences only in insulin and HOMA-IR. *Cyp2j4*^−/−^ rats showed relatively higher glucose and insulin levels, resulting in a higher HOMA-IR index under CAF (Figure 2B). In addition, TAG and NEFA levels were significantly increased in *Cyp2j4*^−/−^ (CAF) when compared to WT (CAF) (Figure 2B).

We then compared the adipocyte area in WAT and subcutaneous adipose tissue (SAT) and found greater numbers of larger and irregularly shaped adipocytes and SAT thickening in *Cyp2j4*^−/−^ (CAF) rats (Figure 2C,D). A lower cell density was observed in *Cyp2j4*^−/−^ (CAF) rats when compared with WT (CAF) rats (Figure 2E) confirming adipocyte hypertrophy in these animals.

### 3.3 Macrophage infiltration and early fibrosis in *Cyp2j4*^−/−^ rats' WAT upon CAF

**Figure 3:**
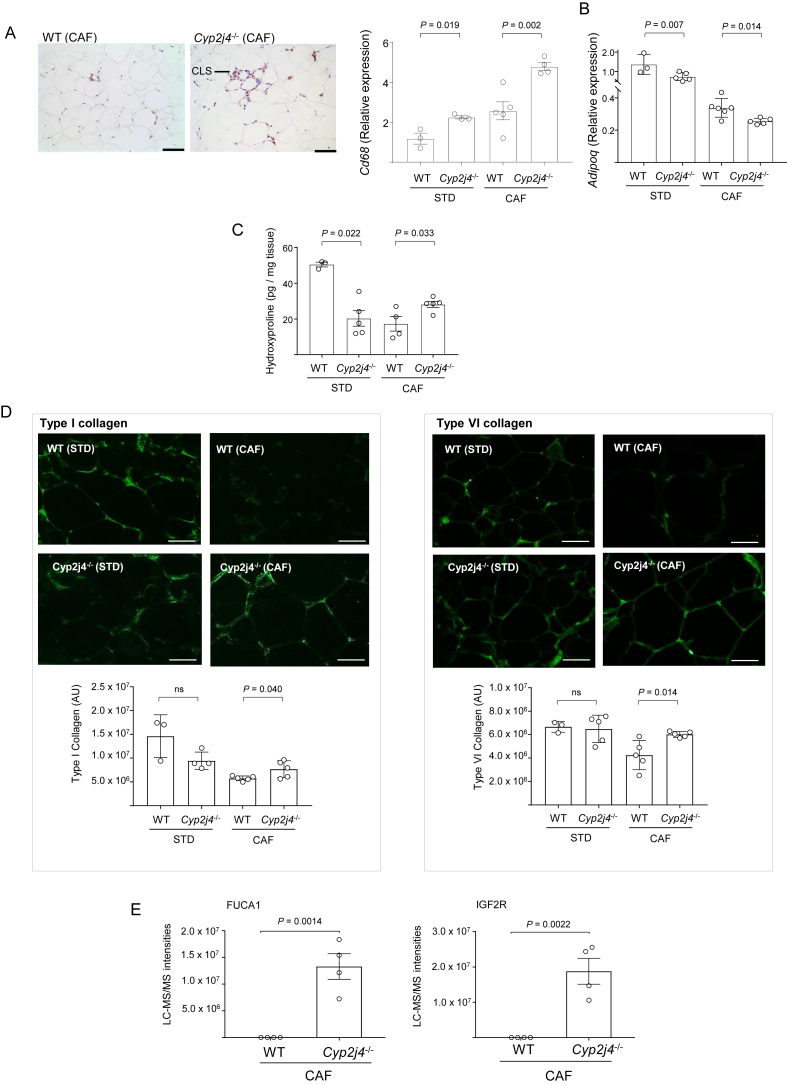
Macrophage infiltration and early fibrosis in *Cyp2j4*^−/−^ rats WAT upon CAF. (A) CD68 (rat ED-1; CLS denotes Crown-like structures) staining in WT (CAF) and *Cyp2j4*^−/−^ (CAF) and qRT-PCR for *Cd68* and *Adipoq* (B) in STD or CAF-trated WT and *Cyp2j4*^*−/−*^ rats. (C) WAT hydroxyproline levels in STD or CAF-treated WT and *Cyp2j4*^*−/−*^ rats. (D) Representative immunofluorescence images for type I (left panel) and type VI (right panel) collagens and their quantification (bottom). (E) LC-MS/MS quantification of FUCA1 and IGF2R, two HIFa targets (TF-binding enrichment) in the stromal vascular fraction of WT and *Cyp2j4*^*−/−*^ rats under CAF. Error bars are s.e.m. At least n = 3 rats were used in each group. ns, non-significant. Scale bars, 100 μm (A) and 50 μm (D).

During adipose tissue expansion, early fibrosis can lead to the infiltration of macrophages, resulting in a chronic inflammatory response [39]. We found relatively increased CD68 positive macrophages organised in Crown-like structures (CLS), and increased Cd68 mRNA levels in *Cyp2j4*^−/−^ WAT (Figure 3A). Adiponectin mRNA levels were significantly reduced in *Cyp2j4*^−/−^ (CAF), suggesting a dysfunctional WAT (Figure 3B). To assess ECM remodeling, we first measured the total hydroxyproline content in WAT. We found relatively decreased hydroxyproline levels in *Cyp2j4*^−/−^ rats under STD diet (Figure 3C), which is in line with the previously observed down-regulation of collagens in MSc-derived adipocytes (Supplementary Figure A.1). Importantly, under CAF, there were significantly higher hydroxyproline levels in *Cyp2j4*^−/−^ rat when compared with WT (Figure 3C). Because the major collagen components in WAT are type I and type VI collagens [37], we next measured these specific collagen types and found significantly higher levels of type I and VI collagens in *Cyp2j4*^−/−^ rats under CAF (Figure 3D). Since adipose tissue hypoxia has been tightly linked to inflammation and fibrosis in metabolic syndrome [38], [40], we next examined hypoxia markers in the stromal vascular fraction (SVF) by quantitative proteomics using liquid chromatography-tandem mass spectrometry (LC-MS/MS). The results showed an enrichment of hypoxia inducible factor 1 α (HIF-1 α) binding sites (1% FDR, *P* = 0.0112), through translational activation of alpha-l-fucosidase (FUCA1) and insulin-like growth factor II receptor (IGF2R), in *Cyp2j4*^−/−^ (CAF) SVF (Figure 3D). Taken together, the results indicate that *Cyp2j4*^*−/−*^ rats show a down-regulation of ECM remodeling under STD diet. However, upon CAF, macrophage infiltration in the WAT and elevated levels of type I and VI collagens together with markers of hypoxia, suggest an early stage phase fibrosis in *Cyp2j4*^*−/−*^ rats.

### 3.4 Increased *de novo* lipogenesis and gluconeogenesis in *Cyp2j4*^−/−^ livers upon CAF

**Figure 4:**
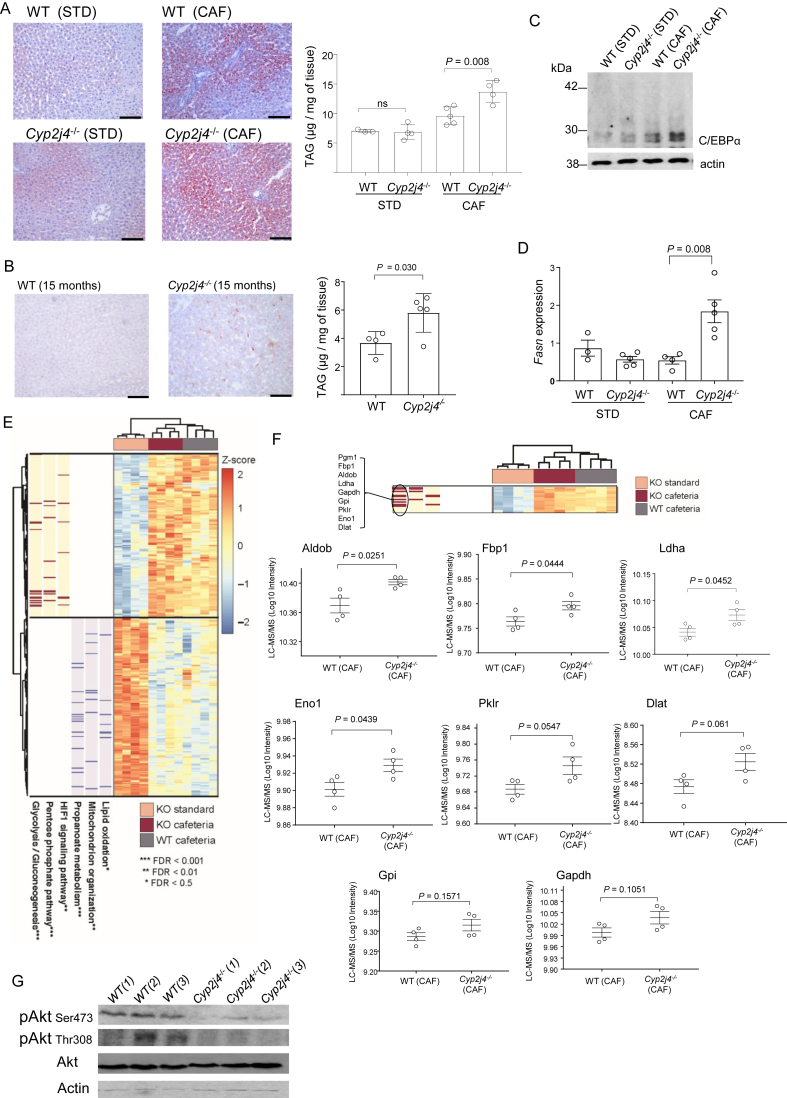
*De novo* lipogenesis and increased gluconeogenesis in *Cyp2j4*^−/−^ livers under CAF. (A) ORO staining and triglyceride (TAG) quantification in STD and CAF-treated WT and *Cyp2j4*^−/−^ rats' livers. (B) ORO staining (left) and TAG quantification in 15-month old WT and *Cyp2j4*^−/−^ livers. (C) C/EBPα Western blot analysis in STD and CAF-treated WT and *Cyp2j4*^−/−^ rats' livers. Blots are representative of 2 independent experiments. (D) qRT-PCR analysis of *Fasn* in STD and CAF-treated WT and *Cyp2j4*^−/−^ rats' livers. (E) LC-MS/MS heatmap displaying the proteins with significant differential protein abundance between *Cyp2j4*^−/−^ (CAF) when compared with *Cyp2j4*^−/−^ (STD) (150 and 164 up- and down-regulated proteins respectively, false discovery rate (FDR) < 0.05). In the heatmap, z-scores of the log-transformed intensities are displayed. Relevant functionally enriched pathways in these two protein sets are shown together with proteins contributing to these enrichments (red and blue bars). (F) LC-MS/MS heatmap (zoomed from E) and protein quantification profiles between WT (CAF) and *Cyp2j4*^−/−^ (CAF) for Pgm1, Fbp1, Aldob, Ldha, Gapdh, Gpi, Pklr, Eno1, and Dlat. (G) Phospho-AKT (Ser473 and Thr308) and total Akt Western blot in CAF-treated WT (n = 3) and *Cyp2j4*^−/−^(n = 3) rats' livers. Numbers denote biological replicates. Error bars are s.e.m. At least n = 3 rats were used in each group. ns, non-significant. Scale bars, 100 μm.

An impaired lipid storage capacity and ECM remodeling of white adipose tissue is related to ectopic fat accumulation, triggering hepatic steatosis. As expected, CAF induced hepatic lipid accumulation in WT and *Cyp2j4*^−/−^ rats as well as increased hepatic TNFα levels (Figure 4A and Supplementary Figure A.3). Consistent with the previously observed early fibrosis in *Cyp2j4*^−/−^ (CAF) WAT, the knockout animals also showed relatively increased hepatic TAGs (Figure 4A). Interestingly, aged *Cyp2j4*^−/−^ rats showed similarly increased hepatic TAG levels (Figure 4B), suggesting that hepatic lipid accumulation is a common feature following CAF and aging when *Cyp2j4* is deleted. In CAF, livers from *Cyp2j4*^−/−^ rats showed relatively increased C/EBPα protein levels (Figure 4C) and fatty acid synthase (*Fasn*) mRNA levels (Figure 4D). To gain insights into metabolic dynamics underlying fatty liver disease in the absence of *Cyp2j4*, we conducted quantitative proteomics analysis by LC-MS/MS in WT (CAF) *Cyp2j4*^−/−^ (CAF) and in *Cyp2j4*^−/−^ (STD) rat livers. This analysis identified 1,436 proteins confidently quantified after filtering (1% false discovery rate (FDR), see also Supplementary Methods A). Differential peptide analysis in the liver showed 150 up-regulated and 164 down regulated proteins between *Cyp2j4*^−/−^ (STD) and *Cyp2j4*^−/−^ (CAF) (Supplementary Table A). Among the up-regulated proteins, the glycolysis/gluconeogenesis, pentose phosphate and HIF signaling pathways showed a significant enrichment (FDR < 0.01) while lipid oxidation, mitochondrion organization and propanoate metabolism were significantly represented pathways among the down-regulated proteins (FDR < 0.05; Figure 4E). When *Cyp2j4*^−/−^ (CAF) and WT (CAF) comparison was considered, the proteins belonging to the glycolysis/gluconeogenesis pathway showed an overall up-regulation in the *Cyp2j4*^−/−^ (CAF) livers (Figure 4F), suggesting a dysregulated hepatic glycolysis/gluconeogenesis. In order to link these results to the corresponding cell signaling pathway, insulin sensitivity in the liver was assessed in *Cyp2j4*^−/−^ (CAF) by comparing phosphorylated levels of Akt with the ones in WT (CAF) (Figure 4G).These results indicate a selective hepatic insulin resistance scenario [41] in *Cyp2j4*^−/−^ animals whereby gluconeogenesis is not suppressed while lipogenesis remains active.

To find out whether the exacerbated hepatic lipid accumulation is associated with hepatic inflammation and/or fibrosis, we have measured total hydroxyproline, type I collagen, and TNFα protein levels but found no differences between *Cyp2j4*^−/−^ (CAF) and WT (CAF) conditions (Supplementary Figure A.3). Liver LC-MS/MS analysis of inflammatory markers (Crp, Eif2ak2, Ikbkg) did not show any significant protein level differences (Supplementary Figure A.3), suggesting that enhanced lipid accumulation did not translate into an increased inflammatory or fibrotic state in *Cyp2j4*^−/−^ rats following 12 weeks of CAF.

### 3.5 *Cyp2j4* deletion causes WAT dysfunction and a shunt in AA pathway following aging and CAF

**Figure 5:**
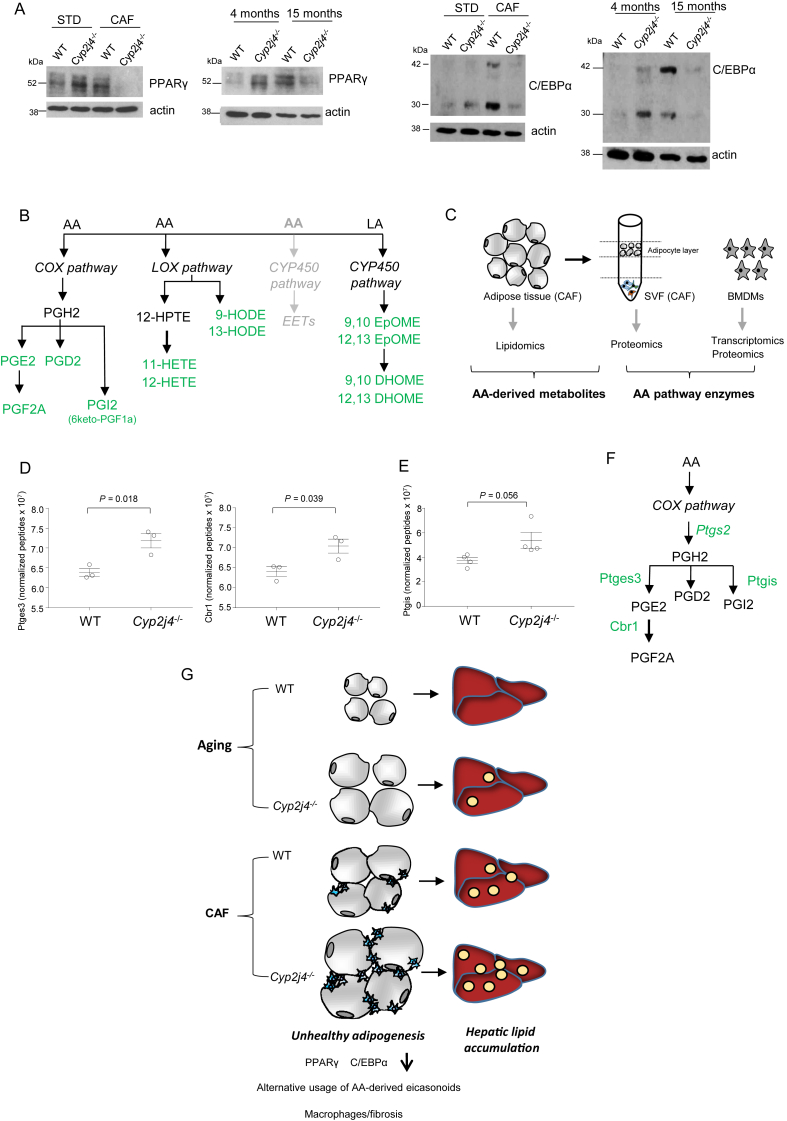
*Cyp2j4* deletion causes WAT dysfunction and a shunt in the AA pathway. (A) PPARγ and C/EBPα Western blot analyses in WT and *Cyp2j4*^−/−^ WAT (STD vs. CAF) or aging (4-month vs.15-month old) conditions. (B) Schematic representation of AA and LA-derived eicosanoids. The Cyp450 pathway is shown in grey to illustrate the inhibition of *Cyp2j4*-derived EET production. All eicosanoids in green are up-regulated either in aging or CAF conditions in WAT from *Cyp2j4*^−/−^ rats. For the quantitative data, see Supplementary Figure A.6A. (C) Schematic illustration of the quantitative lipidomics, proteomics, and RNA-seq datasets obtained from different tissues. The protein and mRNA levels of enzymes responsible for the generation of the eicosanoids detected in (B) were investigated in SVF LC-MS/MS, bone marrow-derived macrophage (BMDM) LC-MS/MS and RNA-seq. (D) Ptges3 and Cbr1 protein levels in WT and *Cyp2j4*^−/−^ BMDMs by LC-MS/MS (n = 3 rats per group). (E) Ptgis protein levels in SVF by LC-MS/MS (n = 4 rats per group). (F) Schematic illustration of the AA COX pathway showing the enzymes that catalyze the synthesis of different prostaglandin species. (G) Graphical summary showing WAT homeostasis under aging and CAF in WT *Cyp2j4*^−/−^ rats. Error bars are s.e.m.

Increased macrophage infiltration, early fibrosis, and presence of markers of hypoxia, when coupled with adipocyte hypertrophy reflect adipocyte dysfunction [42]. To examine whether *Cyp2j4*^−/−^ WAT present characteristics of altered adipogenesis, we evaluated PPARγ and C/EBPα protein levels (Figure 5A) as well as PPARα, PPARβ/δ, LXRα, and FXR (Supplementary Figure A.4). Notably, CAF and aging caused down-regulation of PPARγ and C/EBPα protein levels in *Cyp2j4*^−/−^ rats (Figure 5A), suggesting dysfunctional adipogenesis in WAT, leading to ectopic lipid accumulation in both conditions (Figure 4A,B). When epoxygenase-derived EETs were measured in WAT from *Cyp2j4*^−/−^ and WT rats under a STD diet, LC-MS analysis showed a reduction in all four EET regioisomers (5,6-, 8,9-, 11,12-, and 14,15-EET) as well as a drastic reduction in *Cyp2j4* mRNA levels (Supplementary Figure A.5A). CAF induced a further down-regulation of *Cyp2j4* mRNA and EET levels (Supplementary Figure A.5A).

We next examined the AA-derived eicosanoids other than P450 epoxygenase-derived EETs. LC-MS analysis of hydroxyeicosatetraenoic acids (11- and 12-HETE), hydroxyoctadecadienoic (9- and 13-HODE), Cyp450 linoleic acid (LA) and docosahexaenoic acid (DHA)-derived eicosanoids (9,10- and 12,13-EpOME; 9,10- and 12,13-DHOME, 19,20 EpDPE, 19,20 DiHDPA, 17,18Ep-ETE), COX pathway-derived prostaglandins (6ketoPGF1a, PGE2, PGD2 and PGF2), DHETs, TXB2 and LTB4 showed a general down-regulation of these eicosanoids following CAF (Supplementary Figure A.5B). Importantly, 12 AA and LA-derived metabolites showed a general up-regulation either in aged or in CAF-treated *Cyp2j4*^−/−^ rats (Supplementary Figure A.6A). Figure 5B summarizes all quantified WAT eicosanoids showing an up-regulation in either aging or CAF conditions when *Cyp2j4* is deleted. This indicates alternative usage of WAT AA and LA-derived eicosanoids following CAF or aging in *Cyp2j4*^−/−^ rats. Specifically, LA-derived eicosanoids (DHOMEs and EpOMEs) were more significantly up-regulated in aging whereas the prostaglandins were more significantly up-regulated under CAF (Supplementary Figure A.6A).

Taking into account (i) the preferential up-regulation of prostaglandins in *Cyp2j4*^−/−^ (CAF) WAT (Supplementary Figure A.6A), (ii) the previously described pro-inflammatory properties of prostaglandins [43], and (iii) the increased macrophage infiltration and fibrosis found in *Cyp2j4*^−/−^ (CAF) WAT, we integrated the WAT eicosanoid LC-MS dataset with quantitative proteomics in SVF as well as RNA-seq obtained from WT and *Cyp2j4*^−/−^ basal (unstimulated) bone marrow-derived macrophages (Figure 5C). By doing so, we investigated whether any enzyme in the COX pathway shows differences at mRNA and/or protein level in SVF and/or macrophage fractions. When we screened for all the potential enzymes in the AA and LA pathways, we found that *Ptgs2* is the only COX pathway enzyme in macrophages which showed a significant up-regulation in its mRNA reads upon deletion of *Cyp2j4* (Supplementary Figure A.6B). We further confirmed up-regulation of enzymes involved in the COX pathway both in macrophages and SVF following CAF at the protein level. Ptges3 and Cbr1 were found to be significantly up-regulated in *Cyp2j4*^−/−^ macrophages (Figure 5D) whereas *Cyp2j4*^−/−^ SVF showed a trend for up-regulation of Ptgis after CAF (Figure 5E). Thus, up-regulation of COX pathway enzymes in macrophages could partly explain the increased prostaglandin metabolites in the CAF WAT (Figure 5F). Altogether, these results show that upon CAF, the ‘uncontrolled’ adipogenesis causes an early fibrosis, PPARγ/C/EBPα down-regulation, and an increase in WAT prostaglandins, which are likely to derive from infiltrating macrophages. This dysfunctional adipose tissue is characterized by NAFLD (Figure 5G).

## 4 Discussion

A critical step during the progression from the lean to obese state is the rapid expansion of adipose tissue, which is accompanied by the remodeling of the extracellular network to accommodate the dynamic changes occurring in WAT. Here we report that the rat epoxygenase *Cyp2j4* is critical in adipogenesis and subsequent ECM remodeling. We found that deletion of *Cyp2j4* resulted in spontaneous adipogenesis in MSCs with up-regulation of PPARγ and C/EBPα, two transcription factors that cooperate to allow a wide regulation of adipocyte metabolism [5], [44], [45], [46]. In homeostatic conditions (under STD chow diet), *Cyp2j4*^−/−^ rats also showed relatively increased levels of PPARγ and C/EBPα in their WAT. This genetically determined, enhanced adipogenesis was associated with a decreased type1 collagen and hydroxyproline levels, and in line with the latter, *Cyp2j4*^−/−^ MSCs-derived pre-adipocytes and adipocytes showed a relatively decreased mRNA levels of adipose tissue-related collagens. The inverse correlation between adipocyte expansion and ECM reduction was previously reported [47], and it is now emerging that a looser (or relaxed) ECM may allow for enhanced adipocyte growth and reduced mechanical stress as it was shown for type VI collagen in mice [37], [48]. Interestingly, deletion of *Cyp2j4* resulted in a similar PPARγ up-regulation in bone marrow-derived macrophages but this caused a general up-regulation of ECM-related genes [27], suggesting that up-regulation of PPARγ can have opposite transcriptional effects due to a cell specific epigenetic signatures [49]. It was indeed shown that macrophage-specific PPARγ binding sites are associated with gene silencing in adipocytes [49].

We found that the homeostatic state of accelerated but healthy adipogenesis shifts towards adipocyte dysfunction and increased ectopic lipid accumulation when *Cyp2j4*^−/−^ rats are fed with CAF or left aging on a normal chow diet. In these rats, CAF caused a more pronounced metabolic syndrome with higher degree of obesity and dyslipidemia. The WAT from *Cyp2j4*^−/−^ rats showed features of early fibrosis characterized by significantly higher levels of type I and VI collagens and total hydroxyproline levels. Defining adipose tissue fibrosis requires cautious interpretation as one should take into account *de novo*, pericellular (collagen fibers adjacent to individual adipocytes) ECM deposition, or a defect in degrading existing collagen fibers during adipose tissue expansion [48]. Nevertheless, when combined with increased macrophage infiltration organised in CLS, which often associates with pericellular fibrosis, our results indicate an early stage fibrosis (i.e. mechanical stress resulting from adipocyte hypertrophy) occurring in *Cyp2j4*^−/−^ rats following CAF. Subsequently, increased adipocyte death rate and CLS formation as a result of maximal adipocyte expansion is likely to progress into insulin resistance and liver damage [50], a hallmark of *Cyp2j4*^−/−^ rats fed with CAF.

Our study establishes adipocyte dysfunction in *Cyp2j4*^−/−^ rats after CAF with hypertrophic adipocytes, down-regulation of PPARγ and C/EBPα, and decreased mRNA levels of adiponectin. Hypertrophic adipocytes become dysfunctional and less efficient as metabolic buffers [51]. We observed that adipocytes from *Cyp2j4*^−/−^ rats showed an irregular polygonal shape, characteristic of stressed cells [52], [53]. PPARγ down-regulation and reduced genomic occupancy was previously observed in models of obesity and diabetes [54], [55], [56], [57]. Specifically, the PPARγ-2 isoform prevents lipotoxicity [58] and rare and severely deleterious dominant-negative mutations PPARγ cause pronounced insulin resistance [59]. Here we present that two distinct metabolic stresses with different degrees of intensity (aging and CAF) cause a down-regulation of WAT PPARγ and C/EBPα in *Cyp2j4*^−/−^ rats, which coincides with hepatic lipid accumulation. When considered within a wider clinical context [60], our results suggest that a better fat storage capacity of adipose tissue depending on optimal levels of PPARγ and C/EBPα is essential for reducing the risk of hepatic steatosis.

The exact mechanisms linking *Cyp2j4* deficiency and increased PPARγ levels remain to be elucidated, though prostaglandins could be possible intermediate lipid mediators. In WAT, prostaglandins were down regulated in *Cyp2j4*^−/−^ rats compared with WT under STD, and this trend was reversed under CAF. Intriguingly, this pattern inversely correlated with WAT PPARγ and C/EBPα, while there was a positive correlation with hydroxyproline and type I collagen levels, in agreement with the previously proposed role of PGF (2alpha) signaling in pulmonary fibrosis [61]. These findings support the idea that decreased PPARγ and C/EBPα are related to higher fibrosis and prostaglandin levels in WAT. Given the previously established modulation of PPARγ transcriptional activity by prostaglandins [62], and their well-known involvement in adipogenesis [63], [64], these eicosanoids could be upstream of PPARγ as a result of a crosstalk between the COX and P450 pathways [65], [66]. We found that *Cyp2j4* deletion in basal (unstimulated) macrophages resulted in *Ptgs2* over-expression and in a general up-regulation of prostaglandin producing enzymes in these cells (Ptges3 and Cbr1) and the stromal vascular fraction of the WAT after CAF (Ptgis). These up-regulated COX pathway enzymes in macrophages could be the result of their respective transcriptional activation due to reduced EETs in macrophages as it was previously proposed [66]. The increased prostaglandin levels in the WAT of *Cyp2j4*^−/−^ rats treated with CAF is concomitant with an increased macrophage infiltration. Hence it is likely to be a reflection of macrophage numbers infiltrating WAT yet the basal macrophage COX up-regulation in the absence of *Cyp2j4* suggests that macrophage function is also a contributing factor to WAT prostaglandin levels. Determining this crosstalk between the COX and P450 branches of the AA pathway during adipogenesis require further studies as deleting *Cyp2j4* in macrophages or adipocytes could have different consequences. Furthermore the Cyp2j metabolizing rates have been previously found to be different in male and female rodent hearts [67], suggesting that the AA pathway crosstalk need to be considered in the wider hormonal context. Taken together, our results suggest that a macrophage-derived prostaglandin up-regulation may contribute to WAT dysfunction in *Cyp2j4*^−/−^ rats. Although prostaglandins offer a potential mechanistic link, we cannot exclude an EET-mediated effect on PPARγ transcriptional activity.

EETs have been previously shown to decrease adipogenesis in 3T3-L1 cells [68], and CYP2J2 and EET levels were found to be decreased in human MSC-derived adipocytes when compared with MSCs [69]. Here we show that *Cyp2j4* deletion resulted in ∼50% reduction in all WAT EET regioisomers, suggesting its predominant role in the generation of these eicosanoids. CAF resulted in a drastic reduction in *Cyp2j4* together with EETs and 28 other eicosanoids measured, which confirms the previously established effect of high fat diet on adipose tissue EET production [70]. In keeping with this, human obesity is characterized by a decreased expression of *CYP2J2* in subcutaneous AT [71] and a down-regulation of CYP epoxygenases associate with hepatic insulin resistance in mice [72].

Our study determines the role of *Cyp2j4* and, more generally, AA pathway in adipogenesis by maintaining a balance between pro-inflammatory and anti-inflammatory eicosanoids. We show that *Cyp2j4* has a regulatory role in maintaining healthy adipogenesis through WAT PPARγ-C/EBPα levels and ECM remodeling. The absence of *Cyp2j4* causes adipocyte dysfunction in diet-induced CAF model and, to a lesser extent, in aging. This adipocyte dysfunction associates with lipid accumulation and insulin resistance in the liver. We therefore propose the epoxygenase pathway as a critical checkpoint that could be targeted in obesity-associated hepatic steatosis and insulin resistance.

## Funding

This work was supported by the Medical Research Council (MR/M004716/1 and MR/N01121X/1 to J.B.) and, in part, by the Division of Intramural Research, National Institute of Environmental Health Sciences, NIH (Z01 ES025034 to D.C.Z.) and by a grant from the Spanish government (AGL2014-55347-R to A.A. and M.B.). We acknowledge funding from Advanced Grant ERC-2010-AdG_20100317 (ELABORATE) from the European Research Council (to T.J.A.). M.P. is a Serra Húnter fellow.

## Author contributions

J.B. designed the study. A.O. and X.T. performed most experiments and analyses. J.H.K, C.G.B., M.P., A.A., M.B., and J.B. performed additional *in vivo* experiments. D.C.Z., M.E., and D.B.B. provided lipidomics data and interpreted data. T.J.A. and A.G.D. provided resources and interpreted data. A.M.M. and E.P. performed bioinformatics analyses. J.B. oversaw the study and wrote the manuscript with contributions from X.T. and A.O. All authors discussed and approved the results presented in the manuscript.

## Appendix A Supplementary data

mmc1

mmc2

mmc3
