# Supplementary material for: Epoxygenase inactivation exacerbates diet and aging-associated metabolic dysfunction resulting from impaired adipogenesis
Source: Mol Metab. 2018 Mar 9;11:18–32. doi: 10.1016/j.molmet.2018.03.003 (PMC6001407; doi:10.1016/j.molmet.2018.03.003)
Supplement: mmc2 [file mmc2.pdf]

Supplementary Figure A.1

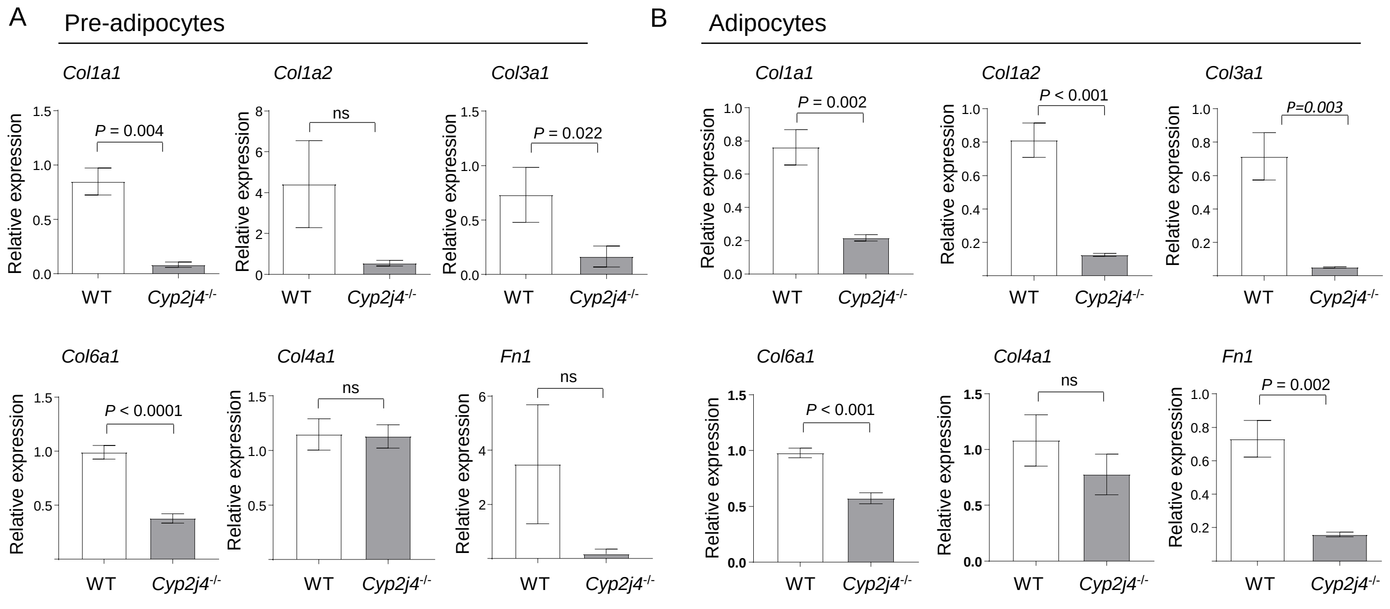

Supplementary Figure A.1. Collagen and fibronectin expression in *Cyp2j4*$^{-/-}$ MSCs-derived pre-adipocytes and adipocytes. qRT-PCR for *Col1a1*, *Col1a2*, *Col3a1*, *Col6a1*, *Col4a1* and *Fn1* in MSC-derived pre-adipocytes (A) and adipocytes (B) isolated from WT and *Cyp2j4*$^{-/-}$ rats. Error bars are s.e.m.

Supplementary Figure A.2

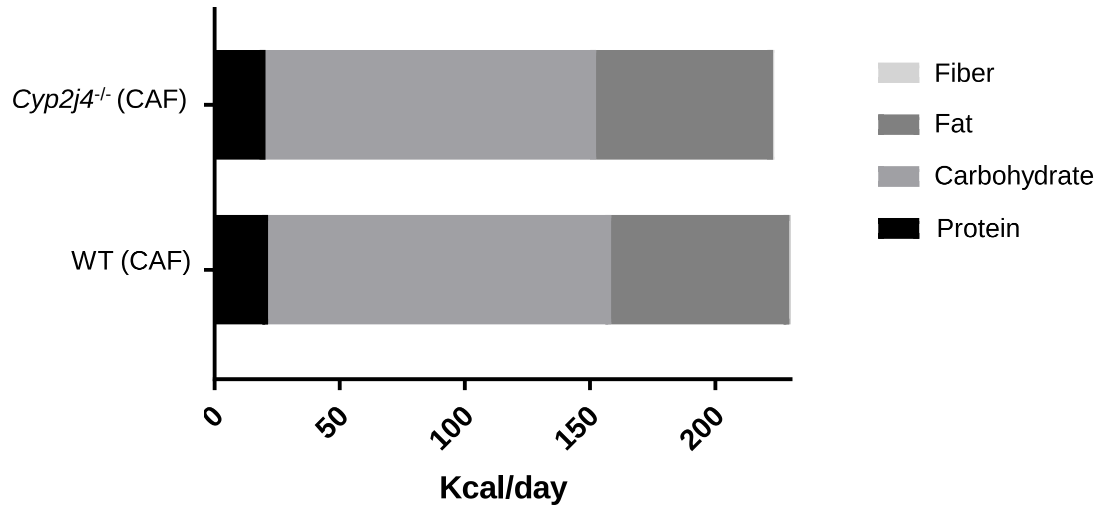

Supplementary Figure A.2. Energy intake is not different between *Cyp2j4*$^{-/-}$ and WT rats under CAF. Kcal / day in fiber, fat, carbohydrates and proteins measured at 11th week following the initiation of CAF.

Supplementary Figure A.3

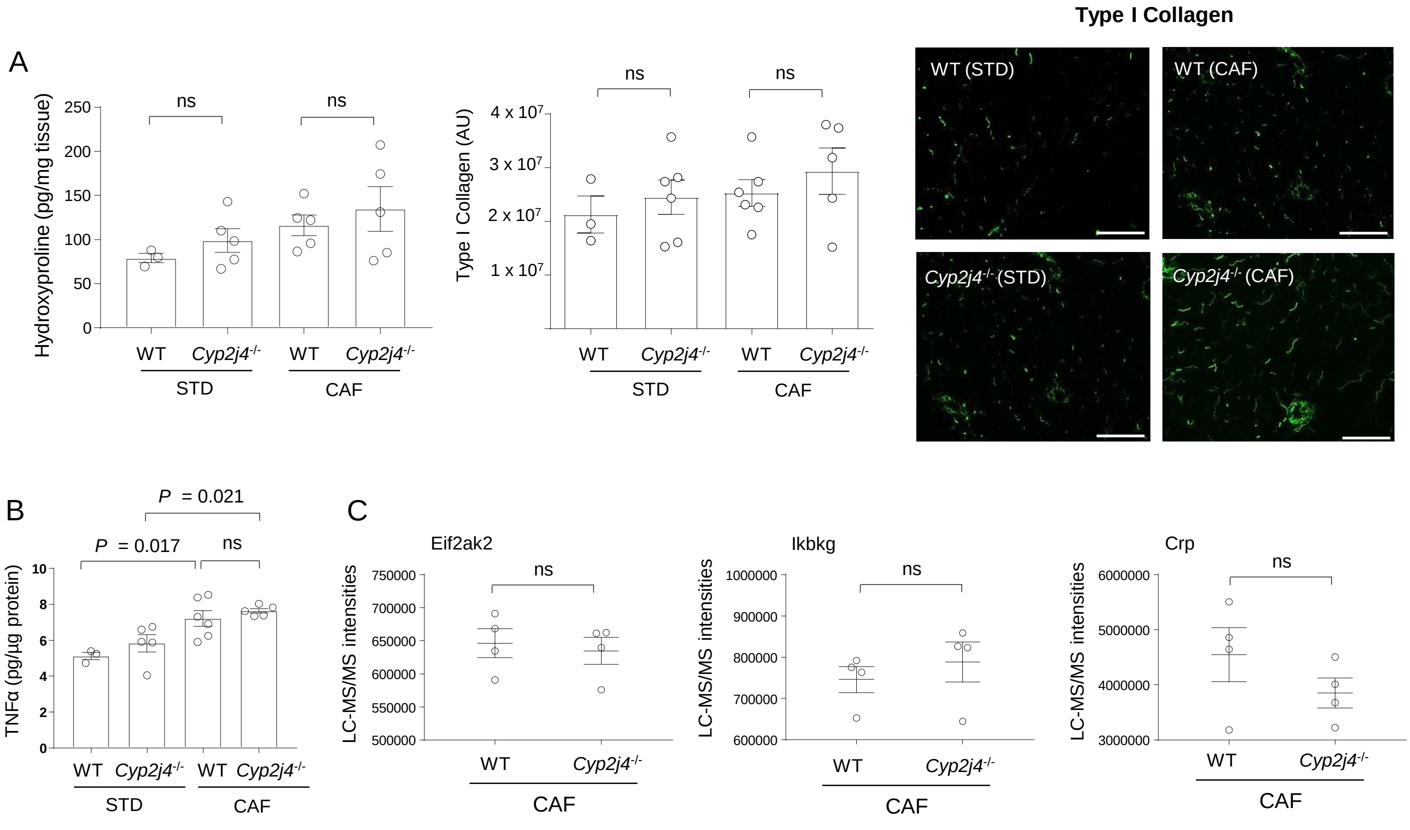

Supplementary Figure A.3. *Cyp2j4-/-* livers do not show increased inflammatory and fibrotic markers upon CAF. (A) Hydroxyproline levels and type I collagen immunofluorescence quantification in WT and *Cyp2j4-/-* livers upon treatment with STD or CAF. (B) TNFα quantification by ELISA in WT and *Cyp2j4-/-* livers upon treatment with STD or CAF. (C) LC-MS/MS profiles for hepatic inflammatory markers such as Eif2ak2, Ikbkg and Crp. At least n=3 rats were used by group. Error bars are s.e.m. AU, arbitrary units; ns, non-significant. Scale bars, 100µm.

Supplementary Figure A.4

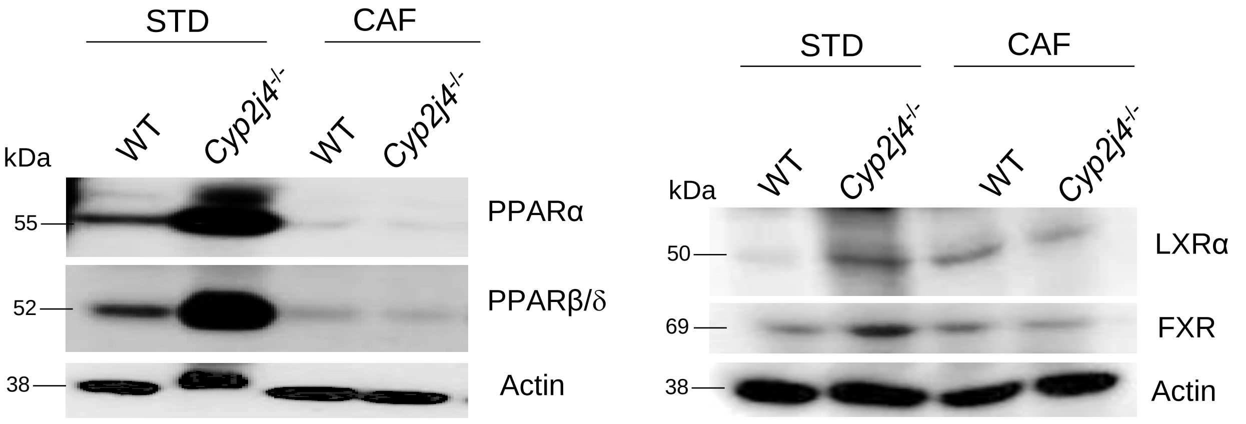

Supplementary Figure A.4. Western blot analyses for PPARα, PPARβ/δ, LXRα and FXR in WAT from WT and *Cyp2j4*$^{-/-}$ rats under STD or CAF conditions.

Supplementary Figure A.5

A

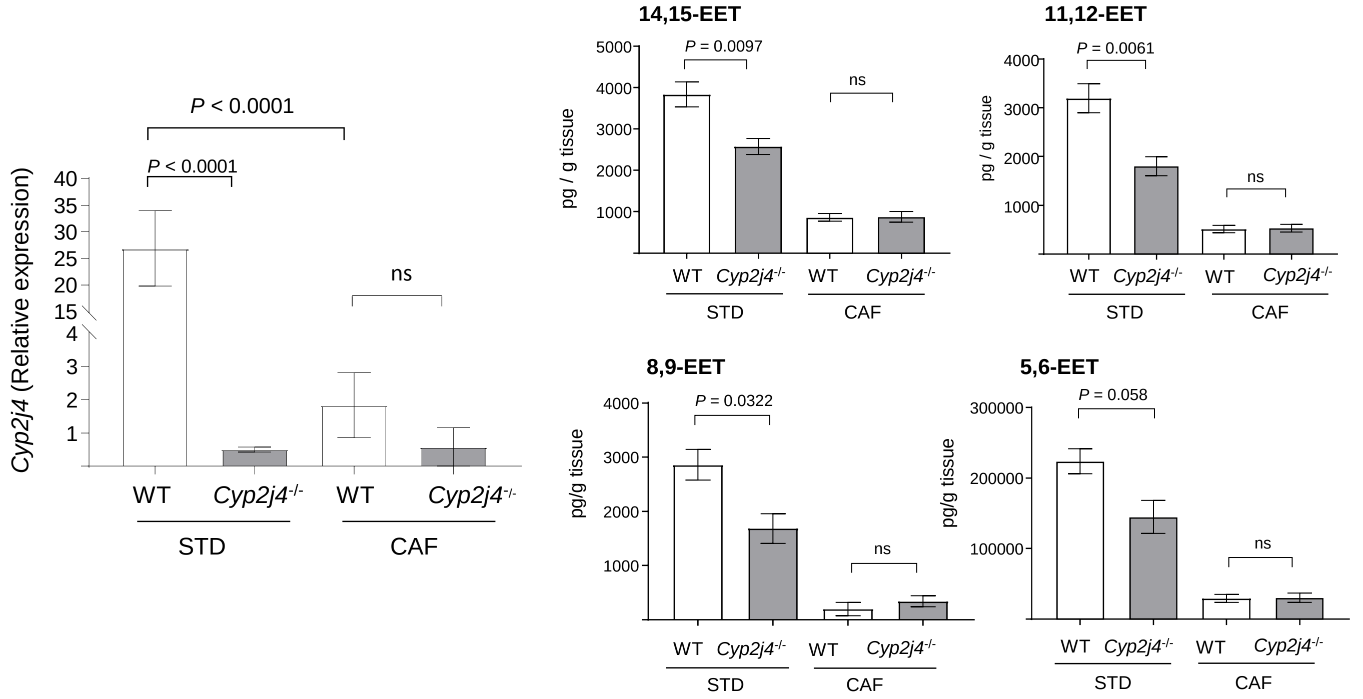

B

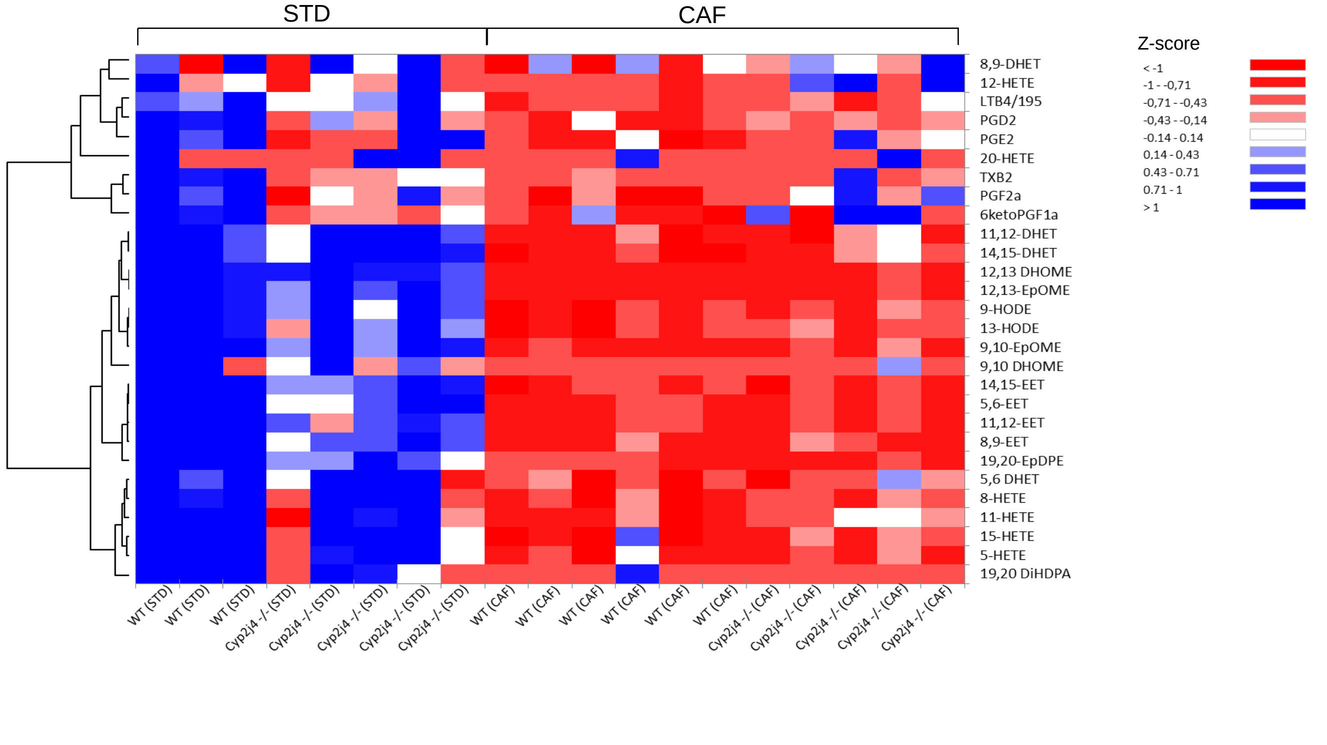

Supplementary Figure A. 5. *Cyp2j4* deletion affects WAT EET and oxylipin levels**.** (A) *Cyp2j4* expression levels measured by qRT-PCR (left panel) in STD and CAF in WT and *Cyp2j4*$^{-/-}$ WAT. The right panel shows LC-MS analysis of 4 EET regioisomers in STD and CAF in WT and *Cyp2j4*$^{-/-}$ WAT. (B) Heatmap showing 28 eicosanoids measured by LC-MS in WT and *Cyp2j4*$^{-/-}$ *WAT* under STD and CAF. At least n=3 rats/group; error bars are s.e.m. ns, non-significant.

Supplementary Figure A.6

A

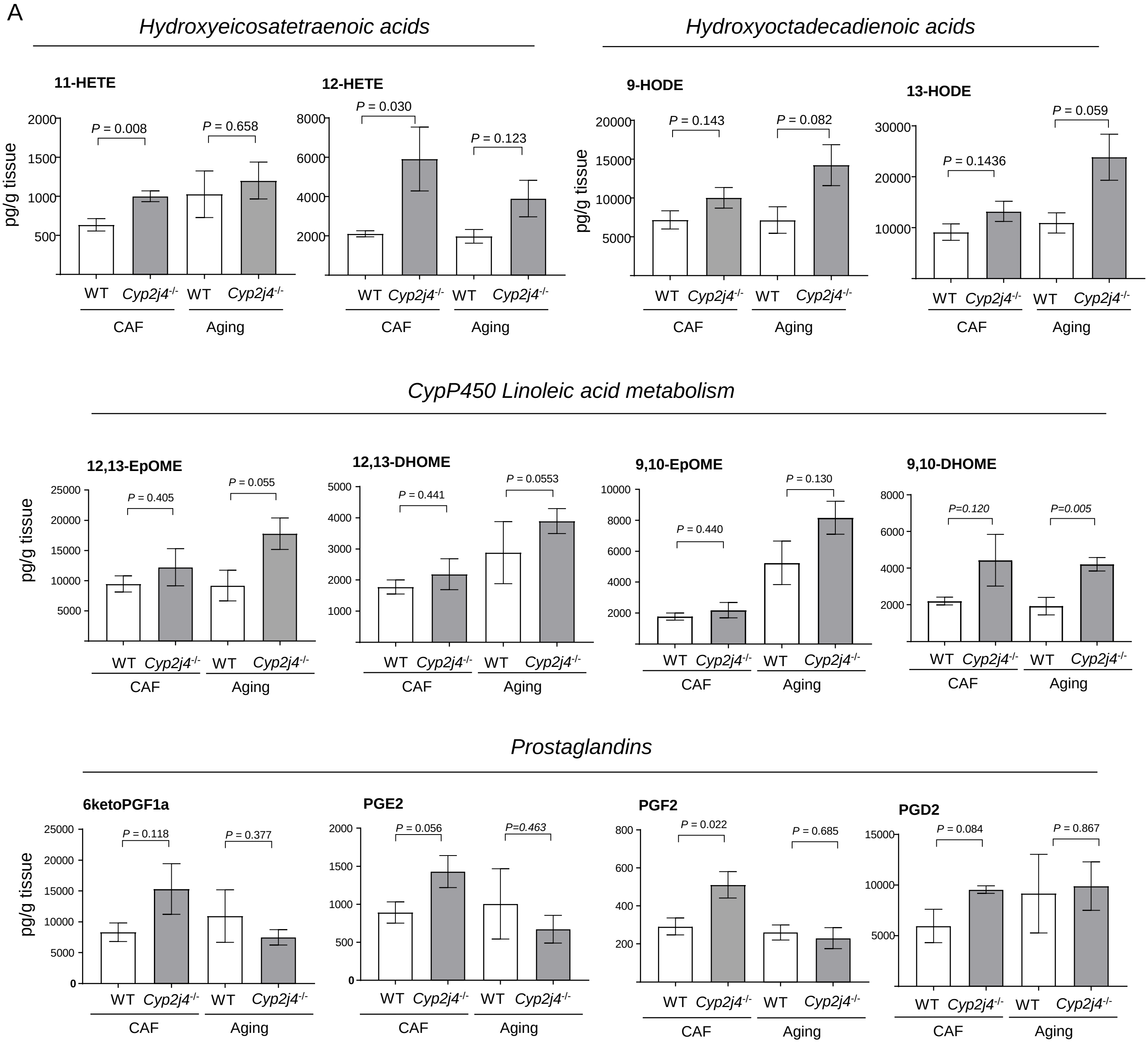

B

Gene	FPKM	LogFC*	*P*	FDR
Cyp2j4	**> 10**	**-4.62**	**5 x 10^{-86}**	**1.51 x 10^{-84}**
Ptgs2	**>1**	**4.15**	**1.62 x 10^{-8}**	**7.36 x 10^{-6}**
Ptges	>10	1.30	0.13	1.00
Ptgs1	>50	-0.08	0.50	1.00
Ptges3	> 300	-0.08	0.51	1.00
Ptges2	>5	0.09	0.61	1.00
Alox5ap	>50	0.22	0.10	1.00
Alox12	>1	-0.11	0.77	1.00
Cyp2d1	>1	0.07	0.77	1.00
Cyp2r1	>1	-0.29	0.26	1.00
Cyp2u1	>1	0.13	0.43	1.00
Cbr3	>1	0.26	0.21	1.00

Supplementary Figure A.6. AA and LA-derived oxylipins are increased in the *Cyp2j4$^{-/-}$* WAT as a consequence of CAF or aging**.** (A) *Cyp2j4* deletion affects WAT HETE, HODE, EpOME, DHOME and prostaglandin levels in either aging or CAF conditions. At least n=3 rats/group. The LC-MS measurements are pg/g tissue for all oxylipins. (B) RNA-seq results in WT and *Cyp2j4$^{-/-}$* BMDMs. FPKM, Fragments Per Kilobase of transcript per Million mapped reads; FC, fold change; FDR, false discovery rate. Error bars are s.e.m.
