# Supplementary material for: Epoxygenase inactivation exacerbates diet and aging-associated metabolic dysfunction resulting from impaired adipogenesis
Source: Mol Metab. 2018 Mar 9;11:18–32. doi: 10.1016/j.molmet.2018.03.003 (PMC6001407; doi:10.1016/j.molmet.2018.03.003)
Supplement: mmc3 [file mmc3.docx]

**Supplementary Methods**

*Morphometric and biochemical variables*

Body weight was monitored weekly and the percentage of weight gain was calculated accordingly. The adiposity index was calculated as ratio between the total adipose tissue weight and total body weight. Plasma glucose (QCA, Spain), non-esterified fatty acids (NEFAs) (Wako, Germany), β-hydroxybutyrate (B-OH) (BEN Srl, Italy) and triacylglycerol (TAG) (QCA, Spain) levels were measured using enzymatic colorimetric kits. Insulin levels were analysed using a rat insulin ELISA (Mercodia, Sweeden). In all assays, the manufacturer’s protocol was followed. Homeostatic Model Assessment for Insulin Resistance (HOMA-IR) index was calculated using the plasma glucose and insulin levels in each animal. For tissue TNF-α and hydroxyproline quantification, protein lysates from liver and adipose tissue were homogenised in RIPA buffer (Sigma-Aldrich) supplemented with 1% Triton X-100 and 1% protease inhibitor Cocktail. Lysates were centrifuged and total protein concentration was determined by Bicinchoninic Acid Kit for Protein Determination (Thermo Fisher Scientific). Tissue TNF-ɑ levels normalized to total protein amount were measured using a rat ELISA kit (BD Biosciences, UK) following the manufacturer’s instructions. Hydroxyproline content (Cell Biolabs, Inc.) was measured in liver and adipose tissue (10 mg of total tissue in each) following the manufacturer’s instructions and expressed as pg/mg of wet tissue weight.

*Food intake*

The rats were housed individually for 24 h, and their food intake was assessed in week 11 of the experiment based on a measurement of the feeding pattern for each animal. The total energy intake and the contribution of each macronutrient to the total intake were determined.

*Adipocyte size and volume quantification*

WAT fragments were fixed in 10% formalin, embedded in paraffin and stained with hematoxylin and eosin (H&E). Stained sections of subcutaneous and epididymal adipose tissues were used to quantify cell areas. Digital images of each section were acquired using an Olympus BX40 microscope (Olympus, UK), and cell areas were traced manually for at least 50 individual cells per field in a blinded manner, using the ImageJ software. Four fields from each section of adipose tissue were analysed in each rat to calculate the mean cell area. Adipose tissue cell number was deducted using the cell volume formula as previously described [[1](#_ENREF_1)]. Briefly, fat cell volume was obtained for each captured field using the formula $\frac{\pi}{6}*\left[ 3\sigma^{2}* \bar{d}+\bar{d}^{3} \right]$ where d is the mean diameter of at least 50 cells, and σ is the standard deviation of the diameter. The mean AT cell weight was calculated from its known density (0.92 g/mL) and cell number in the whole epididymal WAT depot was determined by dividing the total fat depot weight by the mean AT cell weight.

*Primers used in qRT-PCR*

| Gene | Forward | Reverse |
| --- | --- | --- |
| *Cyp2j4* | 5'-AGAGCTTGCCTTGGAGAACA-3' | 5'-GGTGCGTGACTGGAGAAAG-3' |
| *Col1a1* | 5'-AACAAGGGAGGAGAGAGTG-3' | 5'-TGGTGCTCTGAAACCCTGAT-3' |
| *Col1a2* | 5'-TCTAGAAAGAACCCTGCCCG-3' | 5'-GTATGCATTCTTGGCTGGGG-3' |
| *Col3a1* | 5'-ATTGCTGGAGTTGGAGGTA-3' | 5'-GGGTGGCAGAATTTCAGGTG-3' |
| *Col4a1* | 5'-AACATGGTGCTACTCCCTCC-3' | 5'-TTCAGCAAACAGAGGCCAAC-3' |
| *Fn1* | 5'-TGGGAGCGGTTATCTGACTC-3' | 5'-CATTCCCGAGACATGTGCAG-3' |
| *Fabp4* | 5'-TGCAAATTTCCAGTCCAGGGC-3' | 5'-ATGTGCAGAAGTGGGATGG-3' |
| *Fasn* | 5'-TAAGCGGTCTGGAAAGCTGA-3' | 5'-CACCAGTGTTTGTTCCTCGG-3' |
| *Adipoq* | 5′-GGTCACAATGGGATACCGGG-3′ | 5′-GACCAAGAACACCTGCGTCT-3′ |
| *Ppia* | 5′-AGACGCCGCTGTCTCTTTTC-3′ | 5′-ACTTTGTCTGCAAACAGCTCG-3′ |
| *Hprt* | 5′-AAGCTTGCTGGTGAAAAGGA-3′ | 5′-CCGCTGTCTTTTAGGCTTTG-3′ |
| *Cd68* | 5′-TGTTCAGCTCCAAGCCCAAA-3′, | 5′-GCTCTGATGTCGGTCCTGTTT-3′ |

*LC-MS/MS differential protein analysis*

Ensembl v79 was used to map all the proteins to their external gene name using the R library biomaRt version 2.30.0 [2]. Several quality control steps were carried out previous to the analysis of this proteomics dataset: (i) consider only peptides quantified twice or more; (ii) remove proteins for which the two technical replicates did not match (i.e. proteins were detected in only one of the two technical replicates) and compute average peptide intensities across technical replicates; (iii) keep proteins detected in more than half of the biological replicates in at least one experimental condition (i.e. [*Cyp2j4*^-/-^ (CAF)]). This yielded 1,432 proteins in liver (1% Maxquant FDR dataset), 1,194 proteins in SVF (1% Maxquant FDR) and 1,298 proteins in macrophages (1% Maxquant FDR).

Differential protein analysis was carried out using R package limma version 3.30.13 (Ref 2) after log2(data+1) transformation. One pairwise comparison was carried out between “KO with cafeteria diet” (KO_caf) vs “WT with cafeteria diet” (WT_std). Multiple testing correction was set to the Benjamini & Hochberg method (FDR). This resulted in 150 up-regulated and 164 down-regulated proteins in liver significantly with FDR smaller than 5% then comparing KOcaf with KOstd (Supplementary Table 1). The proteins were represented in a heatmap (see Figure 4E) where both columns (samples) and rows (z-score of protein intensities) were clustered by using correlation distance measure and the “ward.D2” method from hclust R function^3^.

Functional and transcription factor binding site enrichment analysis of the differential protein sets in Liver (1% Maxquant FDR dataset) was computed using R package gProfileR version 0.6.1 [3]. We tested the differential proteins with FDR < 5% with positive and negative fold change separately. The background was set to the input set of proteins in the differential protein analysis. External gene names of the differential proteins were used as query and the organism was set as rat ("rnorvegicus"). Electronic annotations were excluded, the p-value correction method was set to “fdr” and results with FDR < 5% were considered. This results can be found in Supplementary Table 1.

**References**

1. Eriksson-Hogling D, Andersson DP, Backdahl J, Hoffstedt J, Rossner S, Thorell A, Arner E, Arner P, Ryden M (2015) Adipose tissue morphology predicts improved insulin sensitivity following moderate or pronounced weight loss. International journal of obesity 39:893-898

2. Ritchie, M.E., Phipson, B., Wu, D., Hu, Y., Law, C.W., Shi, W., and Smyth, G.K. (2015). limma powers differential expression analyses for RNA-sequencing and microarray studies. Nucleic Acids Research 43(7), e47.

3. Juri Reimand, Raivo Kolde and Tambet Arak (2016). gProfileR: Interface to the 'g:Profiler' Toolkit. R package version 0.6.1. https://CRAN.R-project.org/package=gProfileR
